# Is the efficacy of antidepressants in panic disorder mediated by adverse events? A mediational analysis

**DOI:** 10.1371/journal.pone.0178617

**Published:** 2017-06-02

**Authors:** Irene Bighelli, Anna Borghesani, Corrado Barbui

**Affiliations:** 1 Department of Psychiatry and Psychotherapy, Klinikum rechts der Isar, Technische Universität München, Munich, Germany; 2 WHO Collaborating Centre for Research and Training in Mental Health and Service Evaluation, Department of Neuroscience, Biomedicine and Movement Sciences, Section of Psychiatry, University of Verona, Verona, Italy; Carleton University, CANADA

## Abstract

It has been hypothesised that the perception of adverse events in placebo-controlled antidepressant clinical trials may induce patients to conclude that they have been randomized to the active arm of the trial, leading to the breaking of blind. This may enhance the expectancies for improvement and the therapeutic response. The main objective of this study is to test the hypothesis that the efficacy of antidepressants in panic disorder is mediated by the perception of adverse events. The present analysis is based on a systematic review of published and unpublished randomised trials comparing antidepressants with placebo for panic disorder. The Baron and Kenny approach was applied to investigate the mediational role of adverse events in the relationship between antidepressants treatment and efficacy. Fourteen placebo-controlled antidepressants trials were included in the analysis. We found that: (a) antidepressants treatment was significantly associated with better treatment response (ß = 0.127, 95% CI 0.04 to 0.21, p = 0.003); (b) antidepressants treatment was not associated with adverse events (ß = 0.094, 95% CI -0.05 to 0.24, p = 0.221); (c) adverse events were negatively associated with treatment response (ß = 0.035, 95% CI -0.06 to -0.05, p = 0.022). Finally, after adjustment for adverse events, the relationship between antidepressants treatment and treatment response remained statistically significant (ß = 0.122, 95% CI 0.01 to 0.23, p = 0.039). These findings do not support the hypothesis that the perception of adverse events in placebo-controlled antidepressant clinical trials may lead to the breaking of blind and to an artificial inflation of the efficacy measures. Based on these results, we argue that the moderate therapeutic effect of antidepressants in individuals with panic disorder is not an artefact, therefore reflecting a genuine effect that doctors can expect to replicate under real-world conditions.

## Introduction

In randomised studies allocating patients to antidepressants or placebo it is possible that differences in efficacy may, at least in part, be explained by the emergence of adverse events. In the treatment of major depression the evidence is controversial. A meta-analysis of studies comparing fluoxetine with placebo reported a strong correlation between adverse events and efficacy, and authors supposed that the rating scores for patients allocated to antidepressants might have been amplified when study participants became aware of being allocated to active treatment by experiencing adverse events [1]. A more recent study on antidepressants in depression, however, was not able to replicate this finding [2].

A similar hypothesis may be formulated for antidepressants in the treatment of panic disorder, as patients with panic disorder may be particularly prone to adverse events and these, in turn, may have an impact on efficacy measures. The hypothesis is the following. During the informed consent procedure, patients with panic disorder are told that they may receive placebo and are also informed of the side effects to be expected from the real drug. Hence, when they experience side effects, they are likely to conclude that they have been randomized to the active arm of the trial. In fact, most patients and doctors in clinical trials are successfully able to guess whether the patient has been randomized to drug or placebo ([3]; [4]; [5]; [6]). Since the placebo effect is presumed to be associated with expectancies for improvement, concluding that one has been given the real drug (after experiencing adverse events) ought to enhance the therapeutic response, whereas concluding that one has been given placebo (after not experiencing adverse events) ought to diminish it.

If drug-placebo differences are due to the breaking of blind, then they ought to be associated with the perception of adverse events. The purpose of the study reported here is to test the hypothesis that the effects of antidepressants are correlated with the perception of adverse events and to assess what remains of the drug effect when side effects are controlled statistically. We were able to test this hypothesis performing a mediational analysis.

## Materials and methods

### Search methods for identification of studies

The present study is based on an ongoing Cochrane systematic review and meta-analysis of published and unpublished randomised trials comparing antidepressants versus placebo for panic disorder [7]. We refer to Guaiana et al. for a detailed description of the search strategy and full methodology. Briefly, the Cochrane Collaboration Common Mental Disorder Group Trials Register (CCDANTR) was searched. This register includes relevant RCTs from the Cochrane Central Register of Controlled Trials (CENTRAL) (all years), MEDLINE (1950 to date), EMBASE (1974 to date) and PsycINFO (1967 to date). No language restriction was applied. Reference lists of relevant papers and previous systematic reviews were hand-searched. Moreover, the pharmaceutical companies marketing antidepressants, experts in this field and trial authors were contacted for additional unpublished data.

### Types of studies and interventions

We included randomized double-blind comparisons of antidepressants as monotherapy versus placebo in the treatment of panic disorder. For trials that had a crossover design, only results from the first randomized period were considered.

### Types of participants

Participants were in- and out-patients aged 18 years or older of both sexes, with a primary diagnosis of panic disorder with or without agoraphobia, diagnosed according to the criteria described in the Diagnostic and Statistical Manual of Mental Disorders (DSM) [8] and International Classification of Disease (ICD) [9], or according to any other clinical or standardized criteria adopted by the study authors. In case study eligibility focused on agoraphobia, rather than panic disorder, studies were to be included if operationally diagnosed according to the above-named criteria and when it could be safely assumed that at least 30% of the participants were suffering from panic disorder as defined by the above criteria. We included participants with a concurrent secondary diagnosis of another psychiatric disorder. We excluded participants with a concurrent primary diagnosis of Axis I or II disorders and participants with a serious concomitant comorbid physical disorder.

### Data extraction

Two reviewers (IB and AB) independently extracted data from the included studies, and any disagreement was discussed with a third member of the review team (CB).

For the purposes of the analysis reported here, the following two outcomes were extracted: (a) efficacy, measured as number of patients responding to treatment as defined by the original investigators; (b) adverse events, measured as proportion of patients complaining with any adverse events.

Additionally, the following information was collected using an electronic spreadsheet: year of publication, type of antidepressant, sample size, inclusion of elderly participants, setting of intervention, diagnostic criteria, baseline number of panic attacks per week (as a proxy of illness severity), dose and probability of receiving placebo, rated according to Papakostas and colleagues [10]. Mean doses were converted into multiples of the defined daily dosage (DDD) for each drug by dividing the prescribed daily dosage (PDD) by the DDD (PDD/DDD). This measure is the international unit of drug use approved by the World Health Organisation for drug use studies [11,12].

### Statistical analysis

Initially we investigated whether the proportion of patients experiencing adverse events significantly related to the overall efficacy of antidepressants. A meta-regression analysis was carried out with the *metareg* command in STATA, which performs standard random-effects meta-regression using aggregate-level data. Analyses were adjusted for the following variables: sample size, year of publication, type of antidepressant (SSRI versus any other), antidepressant dose (PDD/DDD), probability of receiving placebo (high versus low), outpatients (yes = 1, no = 0), elderly patients (yes = 1, no = 0), severity at baseline (yes = 1, no = 0) (S1 Table).

As second analytical step, the Baron and Kenny mediational model was applied to investigate whether adverse events mediated the relationship between antidepressant treatment and efficacy [13,14]. These analyses were performed at the study arm level. Therefore, in case of multi-arm trials, each arm was considered separately. We reshaped the database from a wide format, where rows identified comparisons, to a long format, where rows identified treatment arms (S2 Table). This allowed us to define the three variables of the Baron and Kenny approach: antidepressants treatment (predictor variable); responders to treatment (outcome variable); individuals with adverse events (mediator variable). According to Baron and Kenny, a mediating role of a variable exists when four conditions are met: (i) the predictor variable must be significantly related to the outcome variable; (ii) the hypothesized mediator must be significantly related to the predictor variable; (iii) the mediator must be significantly related to the outcome; and (iv) the relationship between the predictor and the outcome must be attenuated when controlling for the mediator [13]. When predictor remains significant when the mediator is controlled for, mediation is deemed to be partial. When controlling for the mediator renders the relationship between predictor and outcome non-significant, mediation is deemed complete.

**Fig 1:**
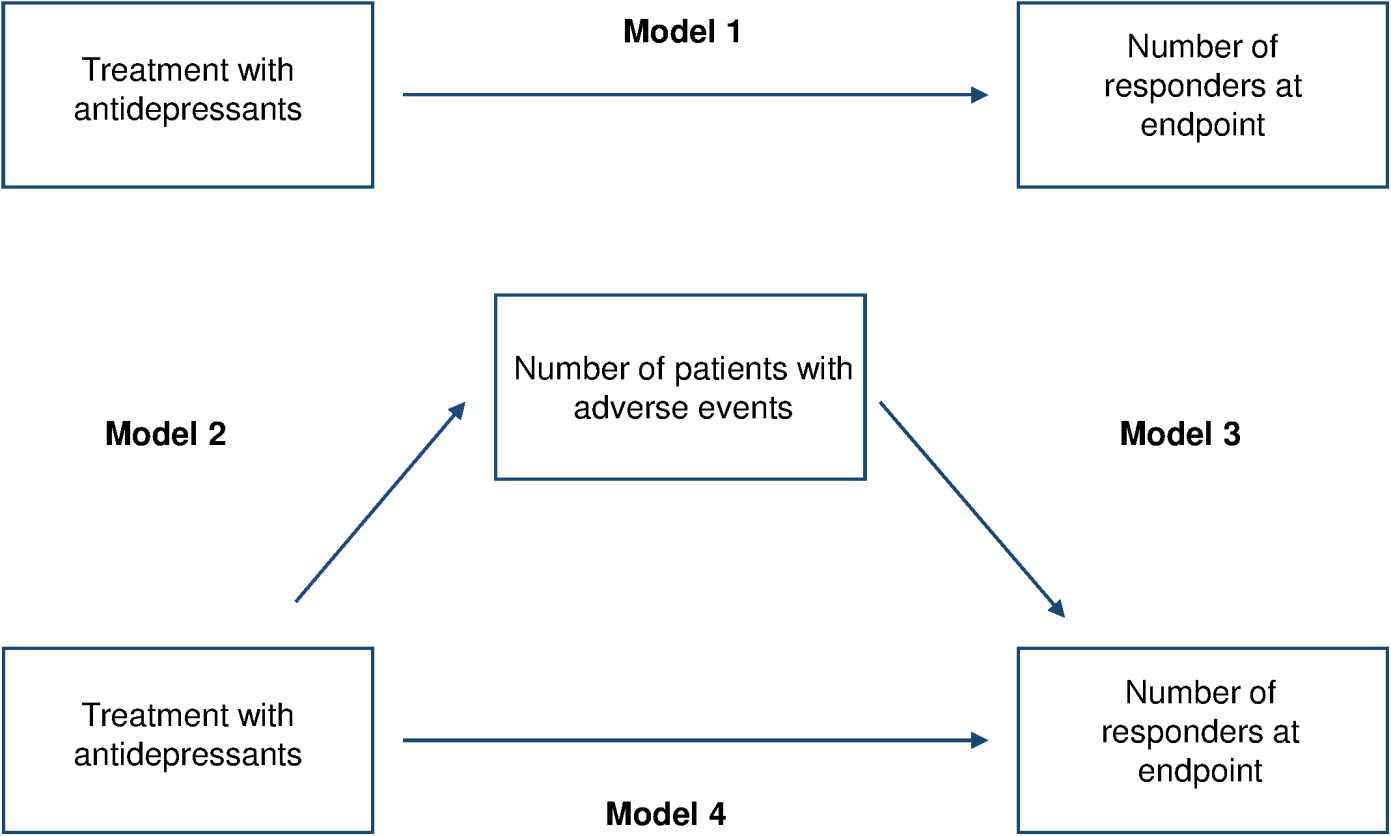
Baron and Kenny mediational model. The model shows both the direct and the mediated pathways by which antidepressants treatment influences efficacy. The mediated pathway investigates the potential mediational role of adverse events in the relationship between antidepressant treatment and the number of responders at endpoint.

A graphical representation of the model applied to antidepressant trials is presented in Fig 1.

In model 1 we tested the relationship between antidepressant treatment and treatment response. In model 2 we tested the relationship between antidepressant treatment and adverse events. In model 3 we tested the relationship between adverse events and treatment response. In model 4 we tested the effect of antidepressant treatment and adverse effects on treatment response. Statistical significance was set at p < 0.05. Mediation was assessed by changes in statistical significance and in the magnitude of correlation coefficients (ß coefficient) between the pathways 1 and 4. To adjust for possible confounding, the four regression analyses included the following variables: sample size, year of publication, probability of receiving placebo, baseline severity, outpatients, elderly patients. A nonparametric bootstrap method of statistical accuracy was used in the four models of linear regression analyses, assuming that the observed distribution of the present sample was a good estimate of the true population distribution [15]. All calculations were performed with Stata13 (STATA Corp, College Station, TX, USA).

## Results

### Characteristics of included studies

**Fig 2:**
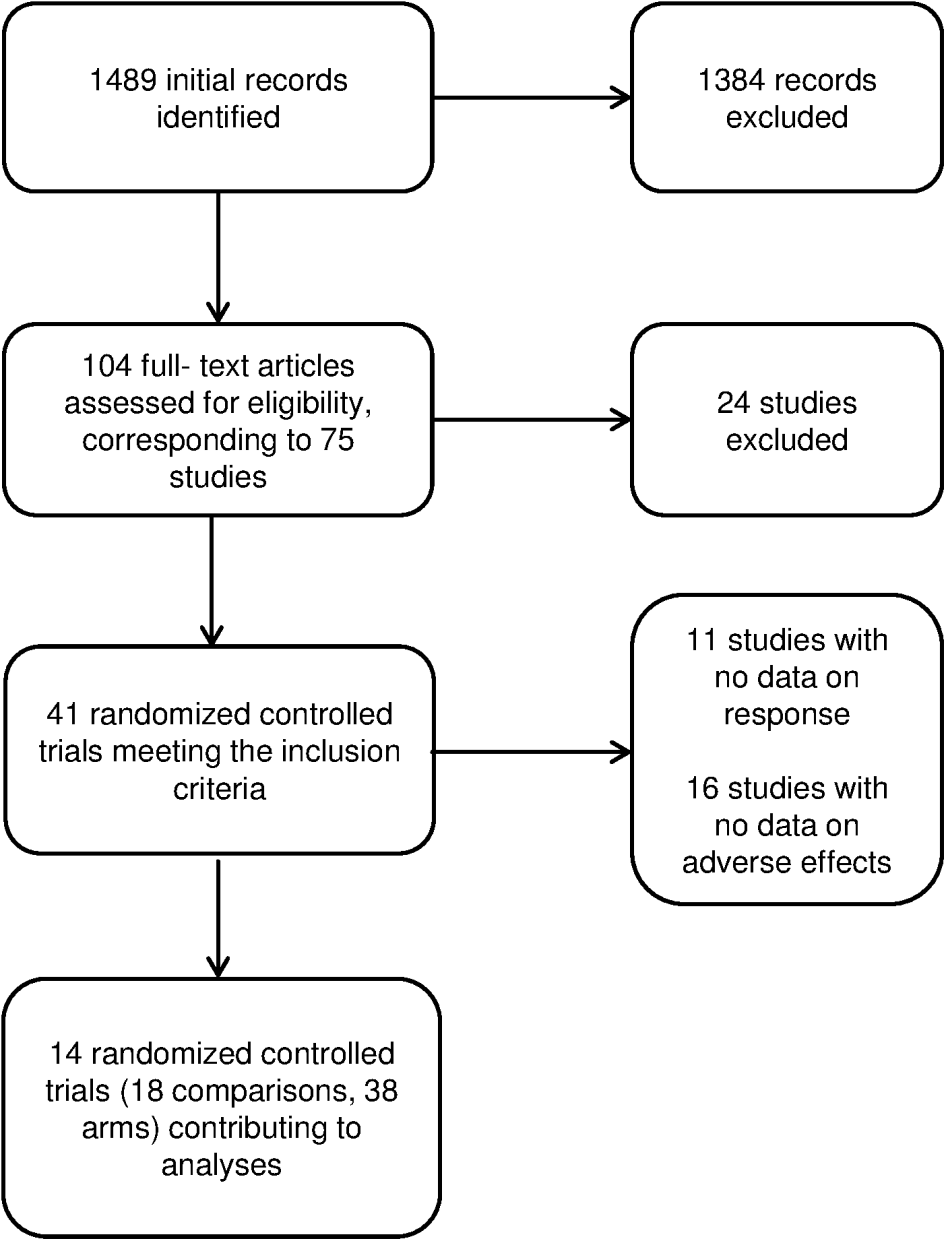
PRISMA flow diagram.

Of the original 41 randomised controlled trials included in the Cochrane review, 11 were excluded because they did not report data on treatment response, and other 16 were excluded because they did not report the number of subjects experiencing adverse effects, leaving 14 studies for the present analysis [16–29]. The study selection process is shown in Fig 2.

Overall, 18 comparisons and 38 treatment arms were considered. The following compounds were investigated in the included studies: imipramine (one study), paroxetine (5 studies), sertraline (3 studies), fluoxetine (one study), fluvoxamine (2 studies), citalopram (one study), escitalopram (one study) and venlafaxine (4 studies). The main characteristics of included studies are shown in Table 1. All studies were double-blind.

**Table 1:** Characteristics of included randomised controlled trials comparing antidepressants with placebo.

STUDY	YEAR OF PUBLICATION	PROBABILITY OF RECEIVING PLACEBO (%)	ANTIDEPRESSANTS	BASELINE SEVERITY*	SAMPLE SIZE		RESPONDERS (%)		ADVERSE EFFECTS		AD DOSE (PDD/DDD)
					Placebo	AD	Placebo	AD	Placebo	AD	
Asnis	2001	50	fluvoxamine	L	92	87	40	54	70	84	2.1
Bradwejin	2005	50	venlafaxine	L	180	181	93	109	138	152	1.6
GSK	1994	33	paroxetine	H	72	77	57	63	58	70	1.7
Liebowitz	2009	50	venlafaxine	L	168	175	87	104	125	144	1.5
Londborg	1998	25	sertraline	L	45	132	18	75	33	111	2.3
Michelson	2001	50	fluoxetine	L	90	90	55	74	19	25	1.4
Nair	1996	33	imipramine, fluvoxamine	H	50	98	13	24	45	93	1.6; 1.7
Pollack	1998	50	sertraline	L	88	88	15	26	77	83	2.3
Pollack-a	2007	25	paroxetine, venlafaxine	H	162	491	94	388	129	413	2.0; 1.5
Pollack-b	2007	25	paroxetine, venlafaxine	H	163	500	87	376	109	367	2.0; 1.1
Stahl	2003	33	citalopram, escitalopram	L	125	255	21	69	94	211	2.1
Tsutsui	1997	33	sertraline	L	56	113	24	50	18	51	2.2
Tsutsui-a	2000	50	paroxetine	L	84	87	27	44	25	42	1.5
Tsutsui-b	2000	33	paroxetine	L	37	83	16	39	13	33	1.2

*Severity: L = low, H = high.

### Univariate and multivariable meta-regression analysis

**Fig 3:**
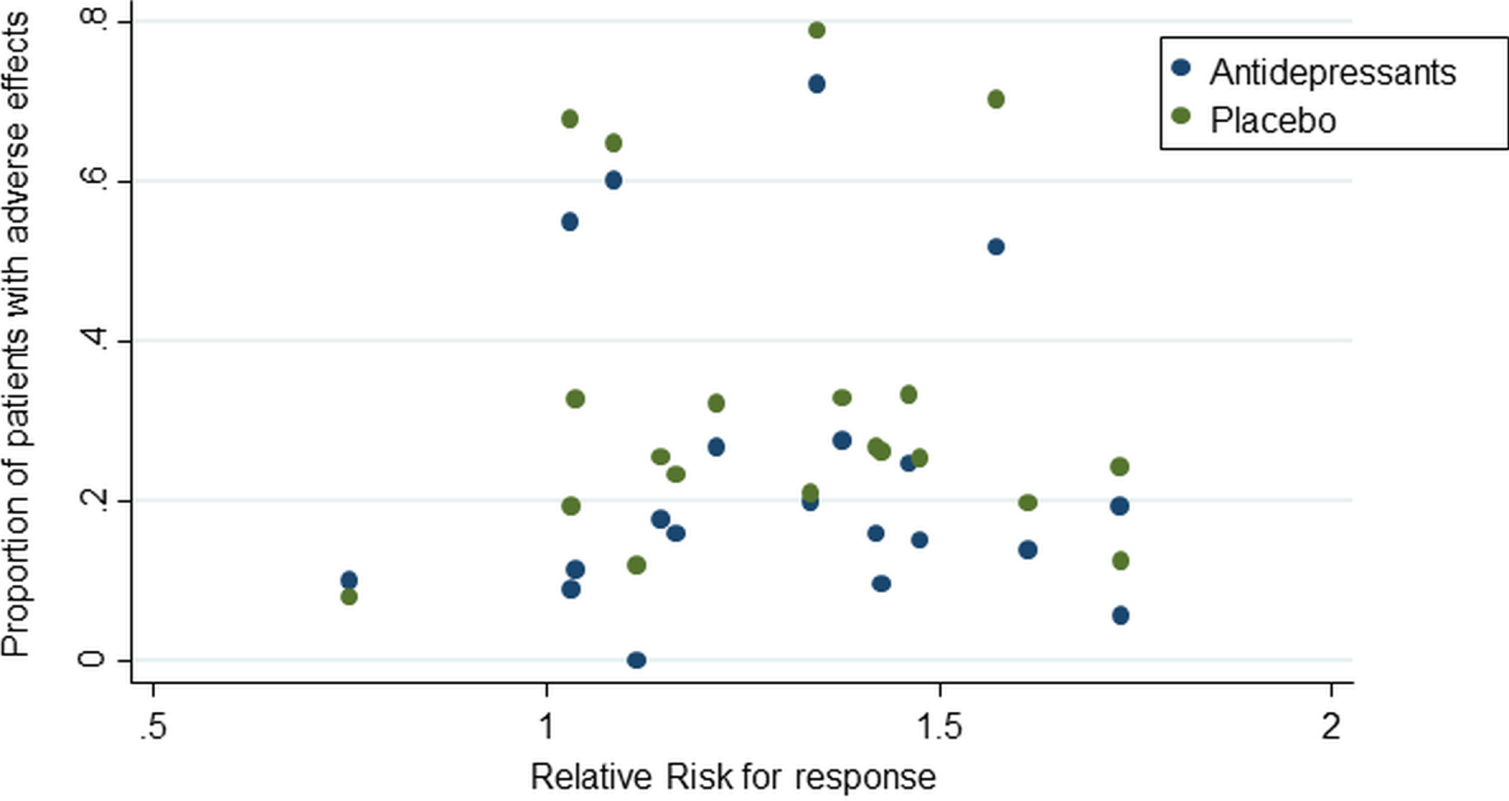
Adverse events and response. Risk >1 favours antidepressants over placebo.

Fig 3 shows that in univariate analysis the RR for efficacy was not associated with the frequency of antidepressant (Spearman rho -0.003, p = 0.990) and placebo (Spearman rho -0.028, p = 0.904) adverse events.

Multivariable meta-regression analysis showed that, after adjustment for potential confounding variables, the RR for efficacy was not associated with the frequency of antidepressant (coefficient -1.752, 95% CI -4.820 to 1.316, p = 0.229) and placebo (coefficient 1.401, 95% CI -1.656 to 4.459, p = 0.327) adverse events (Table 2).

**Table 2:** Meta-regression.

Independent variables	Dependent variable: relative risk AD versus placebo (RR>1 favors AD)		
	Coefficient	95% Confidence interval	P value
**Sample size (continuous variable)**	0.001	-0.002, 0.004	0.379
**Year of study publication (continuous variable)**	0.026	-0.026, 0.078	0.285
**SSRI (yes = 1, no = 0)**	0.379	-0.170, 0.928	0.153
**Dose (PDD/DDD) (continuous variable)**	0.021	-0.398, 0.440	0.912
**Probability of receiving placebo (high = 1, low = 0)**	0.084	-0.282, 0.449	0.617
**Outpatients (yes = 1, no = 0)**	-0.088	-0.629, 0.453	0.721
**Elderly patients (yes = 1, no = 0)**	-0.277	-0.788, 0.234	0.251
**Severity at baseline (high = 1, low = 0)**	0.223	-0.353, 0.799	0.404
**AD adverse effects (continuous variable)**	-1.752	-4.820, 1.316	0.229
**PLO adverse effects (continuous variable)**	1.401	-1.656, 4.459	0.327
**Constant term**	-51.304	-154.614, 52.006	0.290

### Mediational analyses

Table 3 presents the results of the Baron and Kenny approach. Model 1 tested the relationship between antidepressants treatment and efficacy measured as treatment response. In this model, antidepressants treatment was significantly associated with better treatment response (ß = 0.127, 95% CI 0.04 to 0.21, p = 0.003).

**Table 3:** Baron and Kenny mediational model.

MODEL	INDEPENDENT VARIABLE	DEPENDENT VARIABLE	ADJUSTED FOR	β coefficient (95% CI)	P value
**MODEL 1**	Treatment with antidepressants	EFFICACY: NUMBER OF RESPONDERS AT ENDPOINT	Year of publication, probability of receiving placebo, baseline severity, sample size, outpatients, elderly patients	0.127 (0.04, 0.21)	0.003
**MODEL 2**	Treatment with antidepressants	Number of patients with adverse events		0.094 (-0.05, 0.24)	0.221
**MODEL 3**	Number of patients with adverse events	EFFICACY: NUMBER OF RESPONDERS AT ENDPOINT		-0.035 (-0.06, -0.05)	0.022
**MODEL 4**	Treatment with antidepressants	EFFICACY: NUMBER OF RESPONDERS AT ENDPOINT	Same variables as in Model 1, plus number of patients with adverse events	0.122 (0.01, 0.23)	0.039

Model 2 tested the relationship between antidepressants treatment and adverse events. In this model, antidepressants treatment was not significantly associated with higher rates of subjects with adverse events (ß = 0.094, 95% CI -0.05 to 0.24, p = 0.221).

Model 3 tested the relationship between adverse events and treatment response. In this model, adverse events were inversely associated with treatment response (ß = -0.035, 95% CI -0.06 to -0.05, p = 0.022).

Finally, model 4 tested the combined effect of antidepressants treatment and adverse effects on treatment response. When adverse events were added to model 1, the relationship between antidepressants treatment and treatment response was still statistically significant (ß = 0.122, 95% CI = 0.01 to 0.23, p = 0.039), although the effect was slightly attenuated, as can be inferred from the magnitude of the coefficient.

## Discussion

To our knowledge, this is the first study that formally investigated the potential role of adverse events as mediators of treatment effect in antidepressant clinical trials conducted in individuals with panic disorder. We did not find evidence that the effects of antidepressants are mediated by the perception of adverse events.

Our results do not confirm the findings of Greenberg and colleagues [1], who suggested that, in patients with depressive disorder, post-treatment efficacy ratings were inflated by the breaking of blind as a consequence of experiencing adverse events. However, the association between adverse events and improvement reported in the Greenberg et al. meta-analysis was based on four clinical trials only. Additionally, they did not carry out a mediational analysis. In our analysis the approach described by Baron and Kenny was applied, in order to disentangle the potential intermediate role of adverse events as mediators of the relationship between antidepressant treatment and efficacy in individuals with panic disorder. Our findings are consistent with a recent work carried out by Barth and colleagues, who investigated the possible mediational role of adverse events in explaining the efficacy of SSRIs in the treatment for major depression. They similarly found no evidence for a mediational role of adverse events [2].

### Strengths and limitations

Strengths of this study include the following. Data were extracted from an ongoing Cochrane systematic review, which employed a comprehensive search without language restrictions, and included unpublished studies. Standard Cochrane methodology was applied throughout the steps of study selection, data handling and analysis [30]. Although we cannot rule out an even slight effect from unobserved covariates, another strength is that all analyses have been adjusted for a number of study level confounders.

However, a number of limitations should be acknowledged. First, it was impossible to investigate the breaking of blind directly, as the majority of the included clinical trials did not assess this aspect. A second limitation is that only 14 trials out of the 41 potentially relevant studies reported data on the number of participants experiencing adverse events. This is a major issue not only because the analysis might have been more powerful statistically, but also because it is difficult to speculate on whether lack of reporting happened by chance or rather it may reflect some kind of reporting bias. Another limitation is that the meta-regression analysis may suffer from low statistical power. Finally, study quality was not used as a confounding variable as the Cochrane risk of bias tool is considered a qualitative and not quantitative tool [30].

### Study implications

The finding that the drug-placebo differences are not due to the breaking of blind, as there was no relationship with the perception of adverse events, is of paramount relevance clinically and methodologically. Clinically, it implies that the moderate therapeutic effect of antidepressants in individuals with panic disorder is not an artefact, therefore reflecting a genuine effect that doctors can expect to replicate under real-world conditions. Methodologically, it implies that double-blind placebo-controlled antidepressant trials are not biased by the differential emerging of adverse effects in those allocated to the active and control condition, at least in patients with panic disorders.

In terms of implications for research, we argue that strategies to check blindness in future clinical trials should always be employed. This could be achieved by using tools like the one developed by Even and colleagues, who devised a short seven-point checklist to evaluate the degree to which supposedly blind trials are protected from blindness penetration [31]. We also argue that regulatory authorities should compulsory require the routine use of such instruments in clinical trials conducted for regulatory purposes. In fact, any positive changes in terms of trial design, analysis and reporting are likely to be achieved only if they are a requirement of new legislation [32,33]. Similarly, journals editors should no longer accept for publication trials reports that do not include this information.

A different and more radical strategy to overcome this issue would be to avoid placebo-controlled antidepressant trials and require active-control trials in the evaluation of newer antidepressants. In comparative head-to-head trials it is expected that adverse events similarly occur in both treatment arms, with a substantially lower risk of breaking the blind. This seems reasonable in panic disorder, as effective drugs treatments are available in this condition to be used as reference standards [7,34,35]. Regulatory authorities may require active-control superiority clinical trials to generate evidence of superiority between competing treatments [36,33,32,37].

In summary, this analysis does not support the hypothesis that the perception of adverse events in placebo-controlled antidepressant clinical trials may induce patients to conclude that they have been randomized to the active arm of the trial, leading to the breaking of blind and to an artificial inflation of the efficacy measures.

## Supporting information

S1 Table Dataset antidepressants placebo for metaregression analysis. (XLS)

S2 Table Dataset antidepressants placebo for mediational analysis. (XLS)
